# Mechanical thrombectomy of large vessel occlusion using adjustable vs. self-expanding stent-retriever—Comparison of Tigertriever device with stent-like stent-retrievers: A propensity score analysis

**DOI:** 10.3389/fneur.2022.1032307

**Published:** 2023-01-18

**Authors:** Piotr Piasecki, Marek Wierzbicki, Jerzy Narloch, Aleksander Dębiec, Jacek Staszewski

**Affiliations:** ^1^Interventional Radiology Department, Military Institute of Medicine, Warsaw, Poland; ^2^Clinic of Neurology, Military Institute of Medicine, Warsaw, Poland

**Keywords:** mechanical thrombectomy, stroke, Tigetriever, stent-like stent-retrievers, aspiration, Solumbra technique

## Abstract

**Background:**

Stent-retrievers used for mechanical thrombectomy are self-expanding tubular stent-like devices with modified mesh structures for clot removal. Tigertriever is designed to provide manual control of its diameter and curvature.

**Methods:**

A retrospective single-center study was performed to compare Tigertriever with SolitaireX and pRESET (stent-like stent-retrievers group) using propensity score analysis. Patients treated in a comprehensive stroke center due to large vessel occlusion between January 2016 and August 2021 were evaluated. Baseline characteristics and treatment results were compared between these groups before and after pair matching.

**Results:**

There were 140 patients (60 in Tigertriever and 80 in the stent-like stent-retriever group). In propensity score analysis, 52 matched pairs were selected in Tigertriever and stent-like stent-retriever groups. The Tigertriever group had a better successful first pass revascularization rate [46 vs. 23%, OR (95% CI): 1.7 (1.1–2.9), *p* = 0.013] and 14-min shorter groin-to-revascularization time (51 vs. 65 min. *p* = 0.017). There were no significant differences between Tigertriever and stent-like stent-retriever groups in the following: favorable mRS 3 months, favorable recanalization rate, and symptomatic intracerebral hemorrhages. There were no observed periprocedural adverse events related to Tigertriever, SolitaireX, or pRESET.

**Conclusion:**

Tigertriever had a significantly better successful first pass revascularization rate and shorter groin-to-revascularization time in the analysis done before and after propensity score matching with stent-like stent-retrievers. Tigertriever is comparable to stent-like stent-retrievers regarding mortality at 3 months, favorable mRS at 3 months, favorable recanalization rate, or symptomatic cerebral hemorrhagic events.

## Introduction

**Figure 1:**
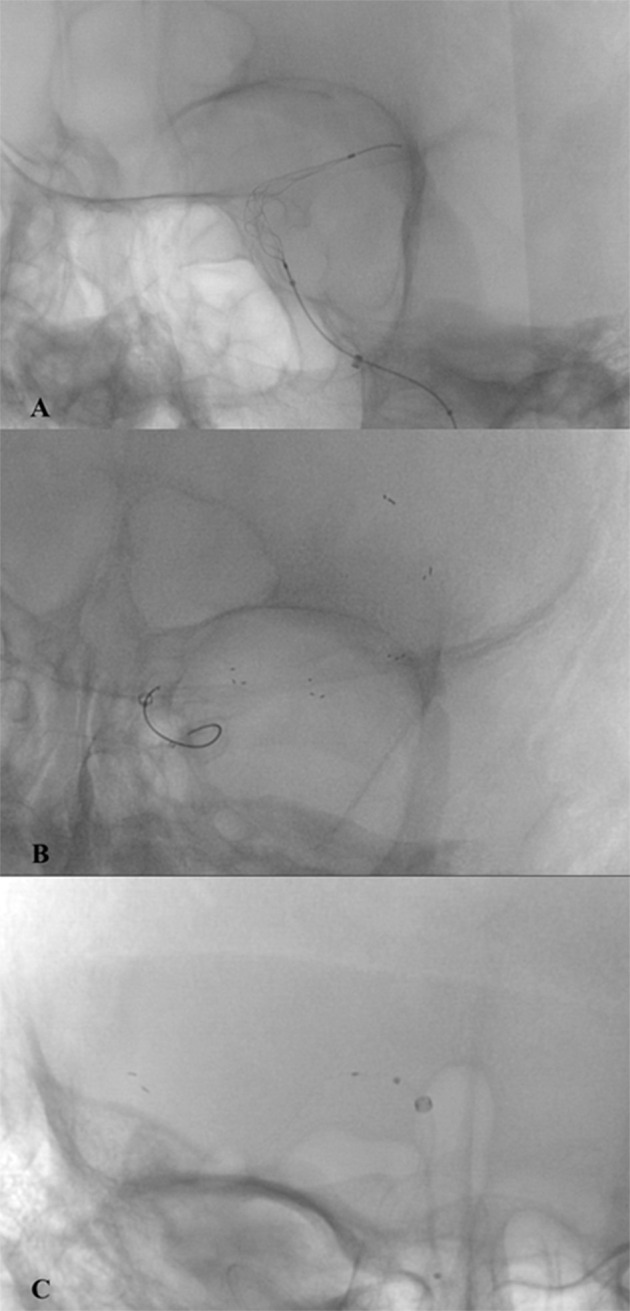
Intraoperative images of SRs during mechanical thrombectomy. **(A)** Tigertriever 17 (Rapid-medical); **(B)** Solitaire × 4 × 40 mm (Medtronic); **(C)** pRESET 4x20 mm (Phenox).

Most stent-retrievers (SRs) used for mechanical thrombectomy (MT) are designed as self-expanding tubular stent-like devices with sophisticated mesh structures allowing better clot integration and removal. Their construction proved its efficacy in large vessel occlusion stroke treatment in many controlled randomized trials published after 2015 (1–5). Examples of stent-like SR devices are SolitaireX (Medtronic), Trevo (Stryker), and pRESET (Phenox, Bochum, Germany) (Figure 1).

SolitaireX is a new-generation thrombectomy device with a closed cell design and a longitudinal split cell section. This SR is fully visible during MT by attached markers (body, proximal, and distal tip). The device is delivered through a microcatheter (inner diameter 0.021–0.027”) *via* a 0.016” nitinol pushwire. SolitaireX is available in multiple lengths: 40, 24, or 20 mm, with diameters of 6 and 4 mm dedicated for MT in large vessel occlusion, and 3 × 20 mm dedicated for distal vessel occlusion.

pRESET (Phenox, Bochum, Germany) is a non-detachable, stent-like construction with a closed ring in the proximal segment and a dual type of SR cell design for stabilizing the structure of the stent. It is produced in three sizes 4 × 20 mm, 5 × 40 mm, and 6 × 30 mm, which work with 0.021” microcatheters—dedicated for MT in case of carotid—T occlusion and middle cerebral artery (MCA) occlusion (6).

**Figure 2:**
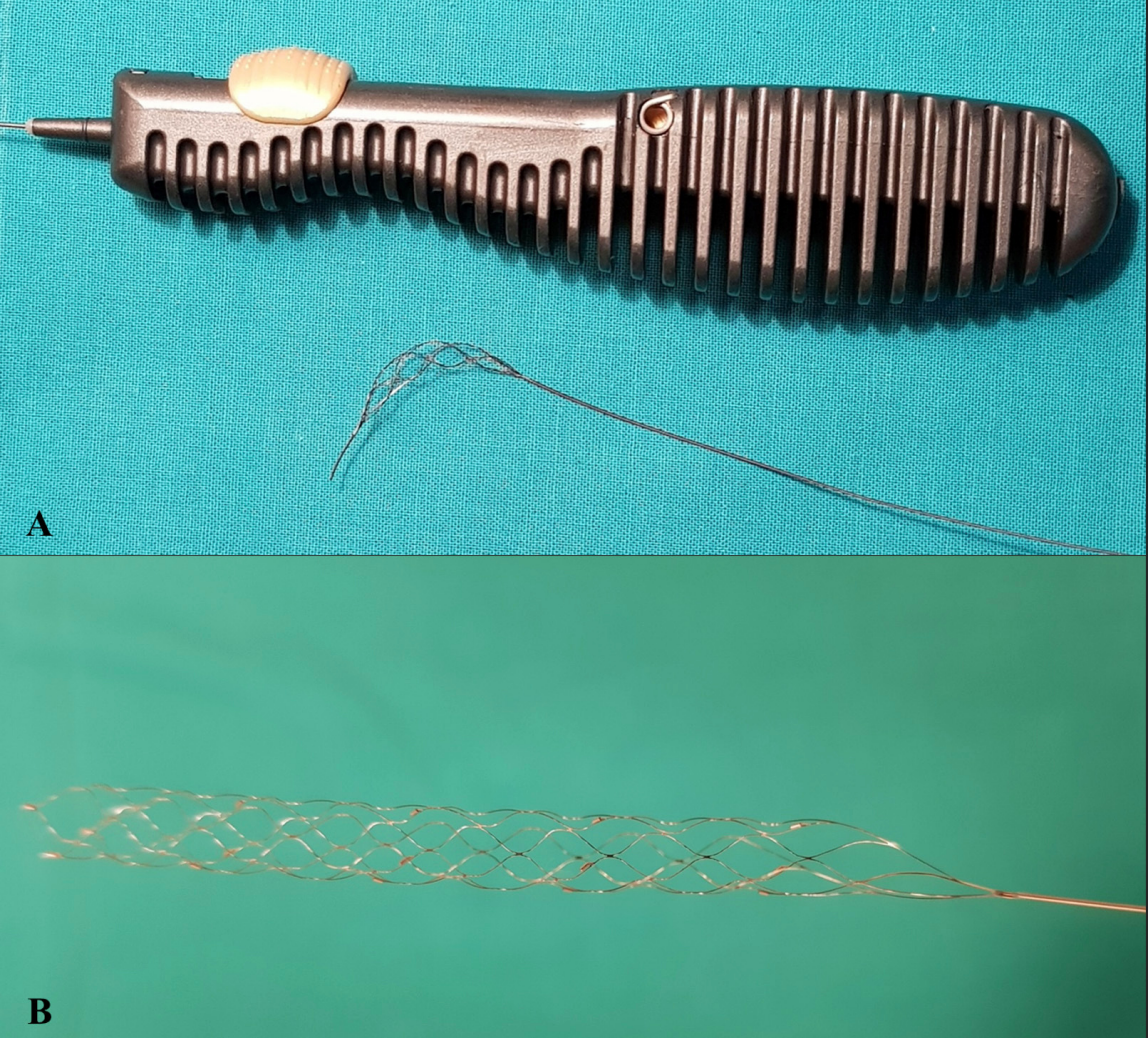
Differences in the structure of devices (Tigertriever vs. Stent-like SR). **(A)** Tigertriever 17 (Rapid-medical); **(B)** Solitaire × 4 × 40 mm (Medtronic).

A different concept was presented by RapidMedical (Israel) with the Tigertriever device (Figures 1, 2). The stent-retriever is fully controlled by the operator by means of a slider on the stent-retriever handle. Device construction enables the operator to adjust the size of the mesh and its radial force to the diameter of the artery and its curvature. Tigertriever is produced in five versions: standard Tigertriever with a net length of 32 mm, which can expand up to 6 mm, is delivered through a 0.021” microcatheter, usually used for MT in larger arteries. Tigertriever 17, with a length of 23 mm, delivered through a 0.017” microcatheter, expanding up to 3 mm. This device is used mainly for occlusions of MCA, anterior cerebral artery (ACA), and posterior cerebral artery (PCA). Tigertriever 13, the smallest version with a net length of 20.5 mm, expanding to 2.5 mm, is used with 0.016” and 0.013” microcatheters and designed for MT in distal vessel occlusion (7, 8). Tigertriver XL is the newest and the largest device in the portfolio. It is designed to remove clots from arteries up to 9 mm in diameter (mostly from an internal carotid artery) (9).

Tigertriever has had a CE mark since 2016 and has shown promising results regarding its efficiency and safety, mostly in single-center studies (7, 10). Tigertriever device proved “to be highly effective and safe” in a recent multicenter study published by Gupta et al., in which it was compared to the data derived from a meta-analysis of six completed trials of Trevo (Stryker) and Solitaire (Medtronic) stent-retrievers (11).

We present a retrospective single-center study, in which treatment results of the Tigertriever device were compared directly with a group of patients treated with Solitaire and pRESET (stent-like stent-retrievers group) using propensity score analysis.

## Methods

### Study design

The goal of this study was to assess the results of large vessel occlusion stroke treatment using a Tigertriever device when compared to Solitaire and pRESET (two stent-like stent-retrievers most often used in our department). The following data were analyzed in a retrospective manner: modified Rankin score at 1 and 3 months, National Institute of Health Stroke Score (NIHSS) before and after stroke treatment, time from onset-to-groin, time from onset-to-recanalization, time from groin-to-recanalization, successful reperfusion rate (mTICI 2b-3), first pass success rate (mTICI 2b-3), and periprocedural complications rate (12, 13).

### Study population

Patients treated in our comprehensive stroke center due to large vessel occlusion stroke using Tigertriever, Solitaire, or pRESET stent-retrievers (stent-like stent-retrievers) between January 2016 and August 2021 were included in the study according to the American Heart Association/American Stroke Association (AHA/ASA) and European Stroke Organization—European Society of Minimally Invasive Neurological Therapy (ESO-ESMINT) guidelines (13–15). Eligible patients received intravenous recombinant tissue plasminogen activator (0.9 mg/kg of rtPA) according to the ESO/AHA guidelines (14, 15).

### Endovascular procedure

The mechanical thrombectomy procedures were done from groin access by operators familiar with assessed stent-retrievers and who had performed at least 50 endovascular stroke treatment procedures. MT was performed under local or general anesthesia at the discretion of the operator and the patient's Glasgow Coma Scale (GCS) status. The operator chose one of the following stent-retrievers to perform thrombectomy: pRESET, SolitaireX, or Tigertriever (mentioned in alphabetical order).

To confirm cerebral large vessel occlusion (LVO), an impress diagnostic catheter (Merit Medical, South Jordan, Utah, USA) was placed in 8F Super Arrow-Flex Introducer Sheath (Arrow Int., PA, USA). The diagnostic catheter was exchanged for a guiding catheter [Neuron MAX (Penumbra, Inc., Alameda, California, USA) or Fubuki (Microvention, California, USA)]. As intermediate catheters, we used: Navien 0.072” (Medtronic, USA), Catalyst 6 (Stryker, USA), React 68 or React 71 (Medtronic, USA), Sophia plus (Microvention, USA), or ACE 68 (Penumbra, USA). Microcatheters: Headway 0.017” or 0.021” (Microvention, California, USA) with 0.014” microwire Traxcess (Terumo, Tokyo, Japan) were used to cross the thrombus. The stent-retriever was deployed at occlusion using the “push and pull” technique. In the stent-like stent-retriever group, the operator waited 5 min to integrate the clot with the stent-retriever mesh. In order to anchor Tigertriever to the thrombus, the “massage” technique was used (repetitive inflation-deflation of the stent-retriever) (7). The Solumbra technique was used during thrombectomy passes. Aspiration passes were done utilizing A Direct Aspiration First Pass Technique (ADAPT) (16).

### Computed tomography (CT) follow-up and study imaging assessment

Non-contrast CT imaging was routinely performed at 24 h after thrombectomy to assess the presence of intracranial hemorrhagic complications. Images were reviewed and evaluated by the two experienced radiologists (PP and MW).

### Statistical analysis

Data are presented as the median for continuous variables and the number with percentage for categorical variables. We calculated mRS and mortality rates at 3 months, mTICI 2b-3 rates at the end of the procedure, first-pass successful recanalization rates, time from onset-to-revascularization, time from door-to-groin, and time of the procedure for Tigertriever and stent-like stent-retriever group. To make a statistical comparison between this group for continuous data, we used Student's *t*-test and Mann–Whitney U test. For categorical variables, chi square or Fisher's exact test was used and a 95% CI of OR was presented. *p-*Values of < 0.05 were statistically significant. We performed propensity score-matched analysis to compare patient groups treated by means of Tigertriever stent-retriever and stent-like stent-retrievers in a 1:1 ratio. The nearest available neighborhood method matching was built based on the data defined by propensity score analysis using a logistic regression model for the following covariates: age, NIHSS at admission, mTICI 2b - 3, and successful first pass recanalization rate (17). We used graphical representations of our data to present the structure of selected variables and to facilitate the comparisons of this group.

## Results

There were 140 patients, who met the study inclusion criteria (60 patients in Tigertriever and 80 patients in the stent-like stent-retriever group). Patients' baseline data are collected in Table 1. In propensity score-matched analysis, 52 matched pairs were selected in Tigertriever and stent-like stent-retriever groups. Baseline characteristics and treatment results were compared between these groups before and after pair matching (Table 1).

**Table 1:** Patients' baseline characteristics and treatment results.

**Characteristics**	**Before propensity score matching**				**After propensity score matching**			
	**All**	**Tigertriever**	**Stent-like SR**	***p-*value**	**All**	**Tigertriever**	**Stent-like SR**	***p-*value**
no. of patients	140	60	80		104	52	52	
**Baseline demographics and medical history**								
Age, median, (years)	71	67	72	0.058^a^	69	68	71	0.405^a^
Men, no./total (%)	64 (46%)	29 (48%)	35 (44%)	0.611^c^	49	25 (51%)	24 (49%)	1.0^c^
Femal no./total (%)	76 (54%)	31 (52%)	45 (56%)	0.611^c^	55	28 (51%)	27 (49%)	1.0^c^
**Medical history, no./total (%)**								
Hypertension	83	47	36	Nd	66	45	21	nd
Atrial fibrillation	45	13	32	nd	38	11	27	nd
Diabetes	25	13	12	nd	19	11	8	nd
Hypercholesterolemia	50	29	21	nd	41	27	14	nd
Current smoking	36	20	16	nd	31	19	12	nd
Coronary artery disease	33	21	12	nd	29	21	8	nd
Previous stroke or transient	16	8	8	nd	9	7	2	nd
**Previous antithrombotic medications**								
- Antiplatelets	44	16	28	nd	33	20	13	nd
- Anticoagulants	38	12	26	nd	28	9	19	nd
**Current stroke event**								
National Institutes of Health Stroke Scale (NIHSS) score on admission, median	15	17	15	0.162^a^	17	16	15	0.593^a^
**Prestroke modified rankin scale score, no./total (%)**								
0	90 (64%)	37 (62%)	53 (66%)	nd	63 (61%)	29 (56%)	34 (65%)	nd
1	31 (22%)	17 (28%)	14 (18%)	nd	27 (26%)	17 (32%)	10 (19%)	nd
2	10 (7%)	3 (5%)	7 (9%)	nd	6 (6%)	3 (6%)	3 (6%)	nd
3	5 (4%)	1 (2%)	5 (6%)	nd	4 (4%)	0	4 (8%)	nd
>3	4 (2%)	2 (3%)	1 (1%)	nd	4 (4%)	3 (6%)	1 (2%)	nd
Intravenous recombinant tissue plasminogen activator, no./total (%)	90 (64%)	35 (58%)	55 (69%)	0.203^b^	64 (61%)	33 (52%)	31 (48%)	0.687^b^
General anesthesia, no./total (%)	57 (41%)	25 (42%)	32 (40%)	0.864 F	42 (40%)	20 (48%)	22 (52%)	0.689^b^
Thrombectomy first line	75	26	49	nd	51	26	25	nd
Rescue thrombectomy after failed aspiration	65	34	31	nd	53	25	28	nd
**Number of passess (median)**								
**No. of patients**								
Aspiration first line	2 (1–5)	2 (1–5)	1 (1–4)	nd	2 (1–5)	2 (1–5)	1 (1–4)	nd
Thrombectomy first line	2 (1–6)	2 (1–5)	2 (1–4)	nd	2 (1–6)	2 (1–6)	2 (1–4)	nd
**Site of occlusion, no./total (%)**								
Middle cerebral artery branch M1	66 (47%)	25 (42%)	41 (51%)	0.210^b^	48 (46%)	22 (46%)	26 (50%)	
Middle cerebral artery branch M2	21 (15%)	15 (25%)	6 (7%)	0.210^b^	19 (18%)	14 (17%)	5 (10%)	
Intracranial internal carotid artery	37 (26%)	15 (25%)	22 (28%)	0.210^b^	26 (25%)	13 (15%)	13 (15%)	
Tandem lesion	10 (7%)	3 (5%)	7 (9%)	0.210^b^	6 (6%)	1 (2%)	5 (10%)	
Basilar artery	6 (4%)	2 (3%)	4 (5%)	0.210^b^	5 (5%)	2 (4%)	3 (6%)	
**Median time frames (min)**								
Onset to groin puncture time, median, min	232	255	217	0.025^a^	240	247	228	0.668^a^
Door (angio-suite) to groin puncture, median, min	32	21	57	< 0.001^a^	30	21	58	< 0.001^a^
Onset to revascularization, median, min	304	305	304	0.089^a^	304	304	304	0.955^a^
Groin to revascularization, median, min	62	57	65	0.029^a^	63	51	65	0.017^a^
**Outcomes**								
Successful revascularization at the end of all procedures mTICI score of 2B/2C/3, no./total (%), OR (95%CI)	84 (60%), 1.2(0.9–1.6)	40 (67%)	44 (55%)	0.163^b^	65 (62%) 1.4 (1.0–2.1)	37 (71%)	28 (54%)	0.068^b^
Successful first pass revascularization mTICI 2B/2C/3, no./total (%), OR (95%CI)	47 (34%), 1.6 (1.0–2.6)	26 (50%)	21 (26%)	0.034^b^	36 (35%) 1.7 (1.1–2.9)	24 (46%)	12 (23%)	0.013^b^
**Clinical efficacy outcomes**								
NIHSS score at discharge, mean	8	8	9	0.605^a^	13	8	17	0.04^a^
Functional independence at 1 month (mRS score of 2 or lower), no./total (%) OR (95%CI)	40 (29%)	21 (35%)	19 (24%)	0.145^b^	31 (30%), 0.5 (0.2–1.2)	19 (36%)	12 (33%)	0.133^b^
Functional independence at 3 months (mRS score of 2 or lower), no./total (%), OR (95%CI)	45 (32%), 1.1 (0.7–1.9)	21 (35%)	24 (30%)	0.531^b^	33 (32%), 0.6(0.2–1.5)	19 (36%)	14 (27%)	0.292^b^
**Adverse events**								
All-cause mortality at 1 month, no./total (%)	38 (27%), 0.6 (0.3–1.1)	12 (20%)	26 (32%)	0.100^b^	28 (27%), 2.7 (1.1-6.8)	9 (17%)	19 (35%)	0.027^b^
All-cause mortality at 3 months, no./total (%)	48 (35%), 0.7 (0.5–1.1)	17 (28%)	31 (39%)	0.199^b^	34 (33%), 2,4 (1.0-5,7)	12 (23%)	20 (38%)	0.089^b^
Symptomatic Intracranial Hemorrhagic Transformation at 24 h after MT, no./total (%):	32 (23%), 0.8 (0.4–1.5)	12 (20%)	20 (25%)	0.486^b^	25 (25%), 1.3 (0.6–3.4)	11 (21%)	14 (27%)	0.491^b^
Subarachnoid hemorrhage, no./total (%):	15 (11%), 0.3 (0.1–1.2)	3 (5%)	12 (15%)	0.095^c^	11 (11%), 3 (0.9-11)	3 (6%)	8 (15%)	0.111^c^
Infarct in new territory no./total (%):	9 (6%), 1 (0.2–4.0)	4 (7%)	5 (6%)	0.920^b^	6 (6%), 1 (0.1–5.0)	3 (6%)	3 (6%)	1.0^b^
Vasospasm no./total (%):	6 (4%), 0.6 (0.1–3.7)	2 (3%)	4 (5%)	0.633^b^	5 (5%), 0.6 (0.1–4.0)	2 (4%)	3 (6%)	0.650^b^
Pneumonia no./total (%):	50 (36%), 1 (0.2–4.0)	21 (35%)	29 (36%)	1.0^b^	42 (6%), 1 (0.2–5.0)	18 (35%)	24 (46%)	0.230^b^

^a^Mann-Whitney U test,

^b^Chi square test,

^c^Fischer exact test. A statistically significant values were highlighted by red color for better data visibility.

### Propensity score matching analysis

**Figure 3:**
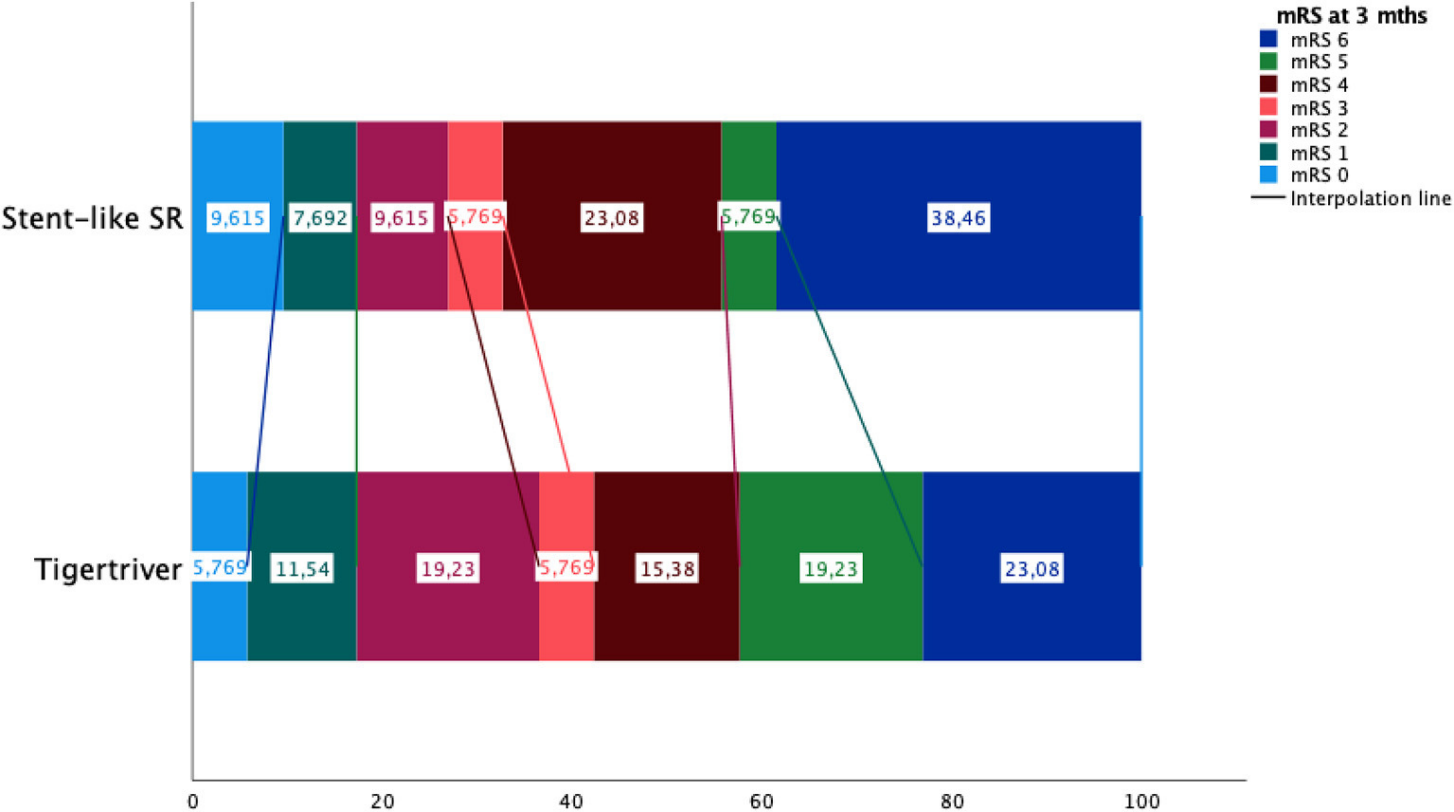
Patients' mRS status after 3 months in Tigetriever and stent-like stent-retrievers group.

Compared to the stent-like stent-retriever group, the Tigertriever group had a better successful first pass revascularization rate [46 vs. 23%, OR (95% CI): 1.7 (1.1–2.9), *p* = 0.013] and 14-min shorter groin-to-revascularization time (51 vs. 65 min., *p* = 0.017). There were no significant differences between Tigertriever and stent-like stent-retriever groups in the following: favorable mRS at 3 months, favorable recanalization rate, and symptomatic intracerebral hemorrhages (Figure 3). There were no observed periprocedural adverse events related to Tigertriever, Solitaire, or pRESET.

### SR performance—ADAPT first vs. Solumbra first

There were 51 out of 104 patients after failed aspiration, and among these, 27 were treated with Tigertriever vs. 24 with stent-like SR. In the first case, successful recanalization was achieved in 18 patients (66%), and in the second case, successful recanalization was achieved in 15 patients (62%), *p* = 0.758. In this group, first pass success was achieved in 48% (13/27) for Tigertriever vs. 4% (2/24) for stent-like SR, *p* = 0.026.

First-line Solumbra treatment was performed in 53 patients, and among these, 25 were treated with Tigertriever vs. 28 with stent-like SR. In the first case, successful recanalization was achieved in 20 patients (80%), and in the second case, successful recanalization was achieved in 13 patients (46%), *p* = 0.031. In this group, first pass success was achieved in 48% (12/25) for Tigertriever vs. 36% (10/28) for stent-like SR, *p* = 0.371.

### Comparison of the Tigertriever and stent-like stent-retriever groups before propensity score matching

The Tigertriever group had significantly a better successful first pass revascularization rate [50 vs. 26%, OR (95% CI): 1.6 (1.0–2.6), *p* = 0.034], shorter groin-to-revascularization time (57 vs. 65 min, *p* = 0.029), and longer onset-to-groin puncture time (255 vs. 217 min, *p* = 0.025). The rest of the baseline and treatment variables were without any significant differences (Table 1).

## Discussion

In this study, we compared the treatment results by manually adjustable Tigertriever stent-retriever and a group of two self-expandable stent-like stent-retrievers (Solitaire and pRESET) using propensity score matching. The latter is a well-known tubular stent-retrievers with proven high efficacy and safety profile (e.g., 77% for TICI>2b for Solitaire device) (6, 18, 19).

We found that the Tigertriever group had a better successful first pass revascularization rate and shorter groin-to-revascularization time in the analysis done before and after propensity score matching with the stent-like SR group. One of the possible explanations behind the reduction of the procedure time in the Tigertriever group may be disposable 5 min needed for clot integration after device deployment as was done in the stent-like SR group. The median time from onset to groin puncture was longer for the Tigertriever group; however, after propensity score matching, the difference was not significant. These could be explained by inter- and intra-hospital transportation times. There were no significant differences between these groups when it comes to mortality at 3 months, favorable mRS at 3 months, favorable recanalization rate, or symptomatic cerebral hemorrhagic events. There is a trend toward a lower SAH frequency for the Tigetriever 3 pts (5%) vs. stent-like stent-retrievers 12 pts (15%) group. This suggests that the Tigertriever's ability to adjust to the diameter of the cerebral artery is not a decisive factor in reducing this type of complication. Moreover, it is believed that SAH in the region of the division of the middle cerebral artery is mainly caused by damage to the perforators to the subcortical nuclei or the insular cortex during thrombectomy.

There are limited studies related to the clinical assessment of the Tigertriever device in patients with stroke. In a few single-center studies done in small patient groups, mTICI 2b - 3 reperfusion rate ranged from 75 to 86% and first pass recanalization rate ranged from 23 to 38% (7, 20). We did not find any research directly comparing Tigertriever devices with stent-like stent-retrievers. However, in a multicenter study done by Gupta et al., 160 Tigertriever treatment results were compared with data from the meta-analysis from six studies, in which Solitaire and Trevo stent-retrievers were most frequently used (e.g., TREVO-2, SWIFT, or MR-CLEAN) (11). Tigertriever achieved a successful reperfusion rate (mTICI 2b - 3) in 84% of patients compared to 63% of other devices set as a performance goal. The first pass successful recanalization rate in the Tigertriever group was 57% compared to 30% in the Solitaire and Trevo groups. Authors concluded that “the Tigertriever device was shown to be highly effective and safe compared to Trevo and Solitaire devices to remove thrombus in large vessel occlusive stroke patients” (11). In regard to our study, we observed mTICI 2b - 3 reperfusion rate of 71% in the Tigetriver group and 54% in the stent-like SR group. The first pass recanalization rate was lower: 46 and 23%, respectively. Our results are similar to Kara et al. reporting on 61 patients treated with Tigertriever, with mTICI 2b - 3 reperfusion rate of 75% and first pass recanalization rate of 34% (7). The data show the high efficiency of the Tigetriver device in the treatment of large vessel occlusion stroke. In our opinion, it may be linked to the device's intrinsic design and construction allowing the operator to fully control the mesh size and radial force during thrombectomy with a slider in its handle. The SR size should be adequately fitted to the cerebral artery diameter, which varies in different segments of the middle cerebral artery and internal carotid artery. For this reason, the stent-like SRs may not fully open in the narrow M2 segment of MCA resulting in poor clot integration with the mesh, while suboptimal vessel wall apposition in relatively wider distal ICA segments could result in clot fragmentation or dislocation leading to stroke in a new territory. Cerebral artery wall apposition during SR retraction is one of the most important factors in the final treatment result and depends directly on its properties (21–23). With a few exceptions, stent-like SR tends to lose their apposition to the inner vessel wall during retrieval when they pass from smaller to wider diameter arteries, and this is linked with loss of radial force (21). Tortuous cerebral arteries create mechanical resistance to stent-retrievers during thrombectomy. The ability to control the Tigertriever size is an advantage in these circumstances, especially, in a sharp vessel, angels often seen between M1 and M2 MCA segments or between ICA and MCA junction (21). Tigertriever can be partially folded on the curve to avoid excessive vessel stretching and damage resulting in subarachnoid bleeding. The operator has limited influence on self-expanding stent-like stent-retrievers after unsheathing in the cerebral artery, with no direct control of its size and wall apposition. An effort to decrease tension on the stent-retriever and vessels can be made by decelerating retraction.

There are limitations to our study. It is a retrospective, single-center study and suffers from a bias inherent to this study design. Due to the relatively small number of individual stent-like thrombectomy cases, we decided to group these into one to be compared with Tigertriever, thus, inter-device comparisons were not possible, and subtle differences could not be appreciable. In this diverse treatment group, we made statistical comparisons based on the first-line treatment, which reflects real-life scenarios; however, we did not emphasize any differences, due to the high likelihood of bias. Further studies are needed to compare Tigertriever with other stent-like stent-retrievers.

## Conclusion

Tigertriever had significantly better successful and first pass revascularization rate and shorter groin-to-revascularization time in propensity score matching with stent-like stent-retrievers. Tigertriever is comparable to stent-like stent-retrievers regarding mortality at 1 and 3 months, favorable mRS at 3 months, favorable recanalization rate, or symptomatic cerebral hemorrhagic events.

## Data availability statement

The raw data supporting the conclusions of this article will be made available by the authors, without undue reservation.

## Ethics statement

The studies involving human participants were reviewed and approved by Ethics Committee of Military Institute of Medicine in Warsaw decision number 34/WIM/2020. Written informed consent for participation was not required for this study in accordance with the national legislation and the institutional requirements.

## Author contributions

Conceptualization and supervision: PP. Methodology, investigation, data curation, and writing—reviewing and editing: PP, MW, JN, JS, and AD. Software: MW, JN, AD, and PP. Validation: PP, JN, and JS. Formal analysis: PP, MW, JN, and JS. Writing—original draft preparation: PP, MW, and JN. All authors contributed to the article and approved the submitted version.
